# Broadly reactive antibodies specific for *Plasmodium falciparum* MSP-1_19_ are associated with the protection of naturally exposed children against infection

**DOI:** 10.1186/1475-2875-11-287

**Published:** 2012-08-21

**Authors:** Arlene E Dent, Ann M Moormann, Christopher T Yohn, Rhonda J Kimmel, Peter O Sumba, John Vulule, Carole A Long, David L Narum, Brendan S Crabb, James W Kazura, Daniel J Tisch

**Affiliations:** 1 Center for Global Health and Diseases, Case Western Reserve University, Cleveland, OH, USA; 2 Pediatrics Department, Rainbow Babies and Children’s Hospital, Cleveland, OH, USA; 3 Pediatrics Department, University of Massachusetts Medical School, Worcester, MA, USA; 4 Kenya Medical Research Institute, Kisumu, Kenya; 5 Laboratory of Malaria and Vector Research, National Institute of Allergy and Infectious Diseases, National Institutes of Health, Bethesda, MD, USA; 6 Malaria Vaccine Development Branch, National Institute of Allergy and Infectious Diseases, National Institutes of Health, Bethesda, MD, USA; 7 Burnet Institute of Medical Research, Melbourne, Australia; 8 Department of Epidemiology and Biostatistics, Case Western Reserve University, Cleveland, OH, USA

**Keywords:** *Plasmodium falciparum*, Antibodies, Merozoite surface protein, Malaria infection, Children

## Abstract

**Background:**

The 19 kDa C-terminal region of *Plasmodium falciparum* Merozoite Surface Protein-1 is a known target of naturally acquired humoral immunity and a malaria vaccine candidate. MSP-1_19_ has four predominant haplotypes resulting in amino acid changes labelled EKNG, QKNG, QTSR and ETSR. IgG antibodies directed against all four variants have been detected, but it is not known if these variant specific antibodies are associated with haplotype-specific protection from infection.

**Methods:**

Blood samples from 201 healthy Kenyan adults and children who participated in a 12-week treatment time-to-infection study were evaluated. Venous blood drawn at baseline (week 0) was examined for functional and serologic antibodies to MSP-1_19_ and MSP-1_42_ variants. MSP-1_19_ haplotypes were detected by a multiplex PCR assay at baseline and weekly throughout the study. Generalized linear models controlling for age, baseline MSP-1_19_ haplotype and parasite density were used to determine the relationship between infecting *P*. *falciparum* MSP-1_19_ haplotype and variant-specific antibodies.

**Results:**

A total of 964 infections resulting in 1,533 MSP-1_19_ haplotypes detected were examined. The most common haplotypes were EKNG and QKNG, followed by ETSR and QTSR. Children had higher parasite densities, greater complexity of infection (>1 haplotype), and more frequent changes in haplotypes over time compared to adults. Infecting MSP-1_19_ haplotype at baseline (week 0) had no influence on haplotypes detected over the subsequent 11 weeks among children or adults. Children but not adults with MSP-1_19_ and some MSP-1_42_ variant antibodies detected by serology at baseline had delayed time-to-infection. There was no significant association of variant-specific serology or functional antibodies at baseline with infecting haplotype at baseline or during 11 weeks of follow up among children or adults.

**Conclusions:**

Variant transcending IgG antibodies to MSP-1_19_ are associated with protection from infection in children, but not adults. These data suggest that inclusion of more than one MSP-1_19_ variant may not be required in a malaria blood stage vaccine.

## Background

Merozoite Surface Protein-1 (MSP-1) is the most abundant protein found on the surface of blood stage *Plasmodium falciparum* merozoites, and has been considered a candidate for a blood stage malaria vaccine. The protein is expressed late in the blood stage cycle as a ~200 kDa precursor protein attached to the merozoite surface via a C-terminal glycosylphosphatidylinositol anchor. Full-length MSP-1 undergoes primary proteolytic processing just prior to schizont rupture, to produce a complex of four MSP-1 fragments that remain non-covalently associated on the merozoite surface [1]. During merozoite invasion of the erythrocyte, a MSP-1_42_ fragment is further processed to produce MSP-1_33_ and MSP-1_19_[1-3]. MSP-1_19_ remains on the merozoite surface during invasion and is readily detectable in newly infected erythrocytes [2]. The *Pfmsp1* gene can be divided into conserved, semi-conserved and variable blocks based on comparisons of deduced amino acid sequences of various clones and field isolates [4]. Block 17 encodes MSP-1_19_ that includes 98 highly conserved amino acids, with the exception of residues 1644 (E/Q), 1691(T/K), 1700 (S/N), and 1701 (R/G). Non-synonymous changes at these positions result in four predominant haplotypes: ETSR (PNG-MAD20 type), EKNG (Uganda-PA type), QKNG (Wellcome type), and QTSR (Indo type) [5-8].

MSP-1_19_ is thought to play a role in erythrocyte invasion as naturally acquired antibodies directed against it can inhibit this process [9-11] and are associated with protection against malaria infection and disease [5,12-19]. However, it is unclear whether protective immune responses are MSP-1_19_ variant-specific or if prior exposure to one infecting haplotype conveys cross protection from another haplotype. Some degree of cross protection has been demonstrated in experimental vaccine studies of *P. falciparum* challenged monkeys [20,21]. Determining the MSP-1_19_ haplotype(s) present during naturally occurring infection is essential for assessment of MSP-1 vaccine efficacy and more generally, studies of variant transcending protective immunity in human populations.

A phase 2 MSP-1 vaccine trial recently conducted in western Kenya showed no evidence of protective efficacy [22]. The vaccine contained 3D7 MSP-1_42_, which includes the ETSR variant of MSP-1_19_. However, the predominant haplotypes in this region have been reported to encode the EKNG and QKNG [23,24], underscoring the potential significance of understanding whether variant-specific immunity is operative. The current study reports the temporal stability of infecting MSP-1_19_ haplotypes among individuals naturally infected with *P. falciparum* malaria in this area, and determines if changes in haplotype were affected by age, infection density, complexity of infection, and pre-existing variant-specific antibody responses.

## Methods

### Study population and design

One hundred and one healthy adults (age range ≥18 to 79 years; average 39.6 years) and 100 healthy children (age range one to 14 years; average 7.7 years) residing in the sub-location of Kanyawegi, Nyanza Province, Kenya were enrolled in a treatment time-to-infection study in July 2003. Malaria is holoendemic in this area, and transmission is relatively high in July. All study participants were afebrile and had normal age-adjusted haemoglobin levels. Venous blood samples were collected at baseline for immunologic and parasite genotyping studies. Witnessed age- and weight-appropriate six-dose regimens of Coartem® (artemether/lumefantrine) were given to all study participants at baseline regardless of malaria infection status determined by blood smear (BS). Weekly finger-prick blood samples were collected for 11 consecutive weeks after treatment. Ethical approval for the study was obtained from the Institutional Review Board for human investigations at University Hospitals Case Medical Center and the Ethical Review Committee of the Kenya Medical Research Institute. Adult participants signed a written consent form in English or Duhluo (the local language); parents or guardians signed in the case of minors <15 years.

### Malaria diagnosis by blood smear

Thick and thin BS were prepared, fixed in 100% methanol, stained with 5% Giemsa, and examined by light microscopy for *P. falciparum*-infected erythrocytes. A slide was deemed negative when no parasites were seen after counting microscopic fields containing at least 200 leukocytes. The density of parasitaemia was expressed as the number of asexual *P. falciparum*/μL blood assuming a leukocyte count of 8,000/μL.

### MSP-1_19_ haplotype detection by PCR/LDR-FMA

DNA was extracted from 200 μL of venous blood and parasite cultures (3D7 = PNG-MAD20 and K1 = Wellcome strains, as positive controls) using QIAamp DNA blood mini kit (Qiagen Corp, Valencia, CA, USA). PCR amplification was performed using MSP-1_19_ specific and *P. falciparum* small subunit rRNA specific primers for 27 cycles (for quantification of parasite density) and 35 cycles (for determination of infection) as previously described [23]. The Ligase Detection Reaction – Fluorescent Microsphere Assay (LDR-FMA) was performed as previously described [23]. Briefly, 1 μL of PCR product (from either 27 or 35 cycle PCR) was ligased with four allele specific probes and two fluorescently labelled conserved sequence probes to detect the four possible haplotypes. Five μL of this LDR was then hybridized to ~250 Luminex® FlexMAP™ microspheres from each allelic set (total number = 5). Reporter streptavidin-R-phycoerythrin (Molecular Probes, Eugene, OR, USA) was added and detection of allele-specific LDR microsphere labelled hybrid complexes was performed using a BioPlex array reader (Bio-Rad Laboratories, Hercules, CA, USA). Each Luminex® fluorescent microsphere emits a unique fluorescent “classification” signal across the range of 658–712 nm. “Reporter” fluorescent signals from R-phycoerythrin are detected, classified into the allele-specific bins, and reported as median fluorescent intensity (MFI) by the BioPlex array reader and BioPlex Manager 3.0 software. Haplotype assignment was made based on allele-specific MFI as described [23]. Importantly, if four alleles (Q, E, KNG and TSR) were detected in a single sample, a conservative assumption was made that only two haplotypes were present. Therefore, the maximum number of haplotypes assigned to any infection was two.

### IgG antibodies to MSP-1_19_ measured by ELISA

IgG antibodies to recombinant PfMSP-1_19_ corresponding to the EKNG, QKNG, ETSR and QTSR variants (expressed in *Saccharomyces cerevisiae* and provided by the Malaria Research and Reference Reagent Resource Center, Manassas, VA, USA [25]) were quantified by ELISA as described previously [26]. Briefly, Immulon 4 plates were coated with 0.1 μg/mL of each MSP-1_19_ protein. Plasma samples from nine North American adults never exposed to malaria were used as the negative controls. Plasma pooled from four known malaria immune Kenyan adults was used to create a standard curve for each plate tested. The value obtained with a 1:50 dilution of the positive pool was designated as 100 arbitrary units (AU), 1:100 dilution as 50 AU, 1:200 dilution as 25 AU, 1:500 dilution as 10 AU, 1:1,000 dilution 5 AU, and 1:2,000 dilution as 1 AU. A four-parameter standard fit curve was constructed from the positive control plasma pool and applied to sample values. Positive values were greater than the mean +3 SD of the value of the individual negative control plasma samples.

### IgG antibodies to MSP-1_42_ measured by Luminex® multiplex assay

Recombinant proteins expressed in *Escherichia coli* were kindly provided by Carole Long and Sanjay Singh (3D7/ETSR and FVO/QKNG) and David Narum (FUP/EKNG) (NIAID, Bethesda, MD, USA). Carboxylated microspheres (Luminex, Austin, TX, USA) were coupled to malaria antigens using the manufacturer’s protocol and as described [27,28]. Briefly, 0.5 μg of recombinant MSP-1_42_ protein was coupled to 6.1 x 10^5^ pre-activated microspheres in 500 μL of 50 mM MES pH 5.0 coupling buffer, vortexed and incubated for two hours at room temperature. Microspheres were washed in PBS, 0.1% BSA, 0.02% Tween-20, 0.05% azide, pH 7.4 (blocking/storage buffer). Antigen-specific IgG was detected by incubating 1,000 beads of each antigen per well with 1:100 plasma dilution in a final volume of 100 uL. After washing, detection with a 1:200 dilution of R-PE-conjugated goat F(ab’)_2_ anti-human IgG antibody (Jackson ImmunoResearch, West Grove, PA, USA) was added. At least 75 beads of each antigen were then acquired by the Bioplex Reader (Bio-Rad, Hercules, CA, USA). Positive and negative controls were as described for MSP-1_19_ ELISA. Results are expressed as MFI and positive values were assigned to samples with an MFI greater than the mean +3 SD of the value of the individual negative control plasma samples.

### MSP-1_19_ invasion inhibitory antibodies (MSP-1_19_ IIA)

Methods to quantify MSP-1_19_ IIA were as described previously [18,29,30]. Briefly, D10-PfM3’ which encodes the MSP-1_19_ MAD20/3D7/ETSR haplotype, and an isogenic D10-PcMEGF parasite line in which the antigenically unrelated murine *Plasmodium chabaudi* orthologue replaces the Pf MSP-1_19_ region were tested in parallel. Ring-stage parasites were synchronized twice by sorbitol lysis and allowed to mature to late trophozoite/schizont stages. Parasites were adjusted to 4% haematocrit with 0.5% *P. falciparum*-infected red cells, and 50 μL aliquots were placed in 96-well, flat-bottom microtiter plates with an equal volume of 1:5 prediluted plasma in culture medium (final plasma dilution 1:10, final volume 100 μL). The same batch of prediluted plasma was added to the two parasite lines in the same assay. The cultures were incubated for 26 hours to allow for schizont rupture and merozoite invasion. Twenty-five μL of resuspended cultures was removed, fixed with 0.25% gluteraldehyde in PBS for 45 minutes, and placed in 1 μg of Hoechst 33342 (HO) stain (Molecular Probes, Eugene, OR, USA) in 400 μL 1x PBS for >24 hours at 4 °C [29,31]. Stained cells were examined using the UV laser on a BD LSR II flow cytometer to collect data from a minimum of 5x10^4^ cells using Becton-Dickinson FACS Diva 5.01. Ring-stage parasitaemia was calculated by quantifying singly infected erythrocytes plus multiply infected erythrocytes (quantified as having two intracellular rings) according to flow cytometry gating previously described [31]. FlowJo 8.5.1 was used to analyse cytometry data. The mean number ring-stage parasitaemia for duplicate wells was calculated and results expressed as a percentage of the ring-stage parasitaemia of non-immune control plasma (derived from non-malaria exposed adults) in parallel cultures. The percentage change of invasion inhibition antibodies specifically attributable to anti-MSP-1_19_ antibodies (MSP-1_19_ IIA) was calculated by subtracting the percentage of invasion of D10-PfM3’ relative to non-immune controls from the percent invasion of D10-PcMEGF relative to non-immune controls. A positive response was defined as ≥10% inhibition attributable to MSP-1_19_ IIA.

### Statistical analysis

Parasite density was compared across groups using the Kruskal-Wallis test. Parasite haplotype distribution across groups was compared using chi square tests and generalized estimating equations. Generalized linear models with robust estimators and exchangeable correlation structure were used to characterize parasite density, frequency of parasite haplotype change over time, and relationship between antibody responses and infecting haplotypes over time. Time-to-infection was compared between baseline variant-specific antibodies (responders *vs* non responders and high levels *vs* low levels) using Kaplan-Meier curves, Wilcoxon and log rank tests. All statistical analysis was performed using Statistical Analysis Software (SAS®) version 9.2 (Cary, NC, USA).

## Results

Analysis of haplotype prevalence, complexity of infection and parasite density was performed using data obtained from all 201 study participants. Data from 25 study participants who were BS negative but positive by the more sensitive PCR LDR-FMA for blood stage *P. falciparum*[23] two weeks after administration of Coartem were presumed to have liver stage infection at baseline. These 25 individuals (three adults and 22 children) were excluded from analyses which compared differences between baseline and follow-up haplotypes with respect to age, *P. falciparum* density, antibody responses, and time-to-infection (n = 176 for these analyses). For clarity, the number of participants analysed is stated with the specific results.

### Prevalence and density of infection by BS and PCR/LDR-FMA (n = 201)

**Figure 1:**
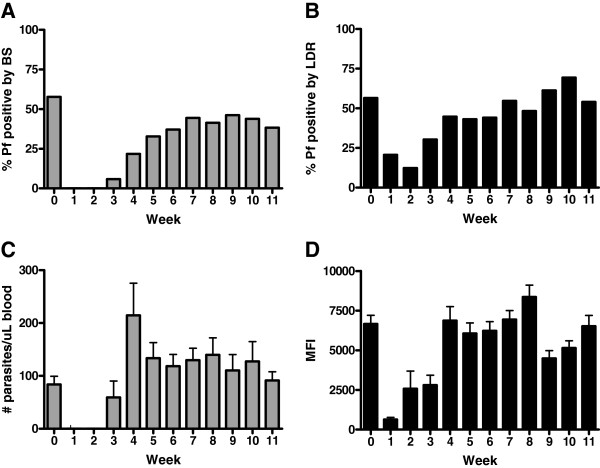
**Prevalence and parasite density by blood smear (BS) and PCR/LDR-FMA.** Panels A and B illustrate the proportion of study participants with infections measured by BS (**A**) and PCR/LDR-FMA (**B**) each week of the study. The parasite density (mean + SD) was calculated from infection positive samples as measured by BS (**C**) and PCR/LDR-FMA (**D**). No statistical difference was detected in BS parasite densities between weeks 3–11, but an increase in parasite densities measured by PCR/LDR-FMA was detected (weeks 3–11; Kruskal-Wallis test, p < 0.0001). The number of infected samples by BS by week: 0 (n = 116), 1 (n = 0), 2 (n = 0), 3 (n = 10), 4 (n = 36), 5 (n = 61), 6 (n = 69), 7 (n = 80), 8 (n = 72), 9 (n = 79), 10 (n = 75), 11 (n = 67). The number of infected samples by PCR/LDR-FMA by week: 0 (n = 112), 1 (n = 39), 2 (n = 25), 3 (n = 52), 4 (n = 73), 5 (n = 81), 6 (n = 83), 7 (n = 99), 8 (n = 85), 9 (n = 103), 10 (n = 118), 11 (n = 94).

Initial malaria prevalence in this population of healthy, asymptomatic individuals was 58% by BS and 57% by PCR/LDR-FMA (Figure 1A and 1B). The proportion of infected individuals by BS remained lower than week 0 (baseline) throughout the subsequent 11 weekly blood samplings. In contrast, the proportion of infected individuals detected by PCR/LDR-FMA showed that baseline infection prevalence was reached after week 7 and stable thereafter. Parasite density was calculated for BS + and PCR/LDR-FMA + samples (Figure 1C and 1D). There was no statistical difference for parasite densities measured by BS after week 2 (weeks 3–11; Kruskal-Wallis test, p = 0.90). However, there was an increase in parasite densities after week 2 when measured by PCR/LDR-FMA (weeks 3–11; Kruskal-Wallis test, p < 0.0001). This most likely reflects the increased sensitivity of PCR/LDR-FMA compared to microscopy. As expected, children at baseline had greater parasite densities than adults (3,740 *vs* 148 *P. falciparum*/μL by BS, p < 0.001; 6,325 *vs* 1,298 MFI by PCR/LDR-FMA, p < 0.001 Kruskal-Wallis test).

### Complexity of infection (COI; n = 201)

**Figure 2:**
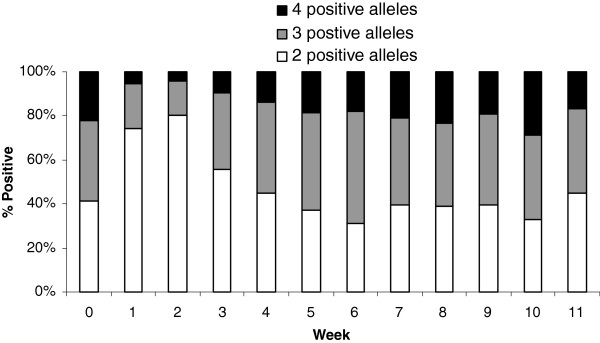
**Proportion of alleles in malaria infected samples reflecting complexity of infection.** Baseline (week 0) distribution of allele frequencies was re-established by weeks 5 (no statistical difference between baseline and week 5).

Block 17 of the *Pfmsp1* gene has four alleles, E, Q, KNG, and TSR, four distinct haplotypes referred to as EKNG, QKNG, ETSR, and QTSR. The proportion of individuals each week that had two, three or four alleles was examined (Figure 2). As expected, when *P. falciparum* density was low because of recent drug elimination, the COI was diminished compared to baseline (before Coartem treatment), dropping from 60% to 24% of infections with two or more alleles. Baseline complexity levels were reached by week 5 and remained relatively stable throughout the remaining study period. Children tended to be infected with more alleles at any given week compared to adults (e.g. 75% of children had >2 alleles at baseline *vs* 34% of adults, p < 0.001).

Generalized estimating equations were used to model single *vs* multiple haplotypes over time according to 1) parasite density at baseline or first *P. falciparum* infection during follow-up; 2) current parasite density; 3) prior (lagged) parasite density; 4) total number of infections within an individual; and, 5) age, while accounting for repeated observations. There was no predictive value found of baseline parasite density for COI. However, individuals with multiple haplotypes at baseline tended to have multiple haplotypes detected during the follow-up period (p = 0.001-0.081). Finally, children were 12.1 times more likely to have multiple haplotype infections compared to adults (95% CI 4.5-32.4; p < 0.001)

### Haplotype prevalence at baseline and follow-up (n = 201)

Of all 964 *P. falciparum* + samples, the majority of individual haplotypes among a total 1,533 was EKNG (n = 736) followed by QKNG (n = 517), ETSR (n = 148) and QTSR (n = 132). There were multiple haplotypes in 570 infections.

**Figure 3:**
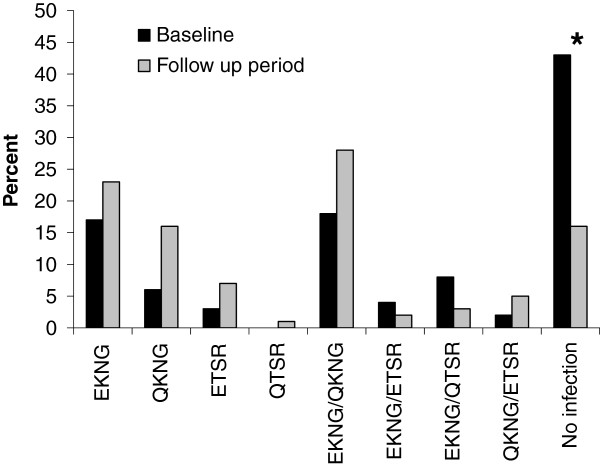
**Prevalence of haplotypes at baseline and the first detectable haplotype during the follow-up period for each study participant.** Baseline prevalence is indicated by black columns and first follow-up period haplotype detected is indicated by grey columns.* indicates statistically significant difference (p < 0.001; Chi-square) between baseline and follow-up proportion of individuals with no infection.

For all subsequent analyses, “no infection” and multiple haplotype combinations (such as EKNG/QKNG, EKNG/ETSR etc.) were included. Figure 3 illustrates the overall prevalence of each haplotype group at baseline and first-detected infection at weeks 3–11 (n = 176). No infection (43%) was most common at baseline followed by EKNG/QKNG (18%), EKNG (17%), EKNG/QTSR (8%), QKNG (6%), EKNG/ETSR (4%), ETSR (3%) and QKNG/ETSR (2%). Similarly, the most frequent haplotype first detected during follow-up was EKNG/QKNG (28%), EKNG (23%), QKNG (16%), no infection (16%), ETSR (7%), QKNG/ETSR (5%), EKNG/QTSR (3%), EKNG/ETSR (2%) and QTSR (1%). There were no statistical differences between the prevalence of baseline haplotypes and haplotypes detected during follow-up. The only significant difference was that the prevalence of “no infections” was lower during the follow-up period compared to baseline (p < 0.001).

Chi-squared analyses were stratified by adult *vs* child to compare baseline *vs* follow-up infecting haplotype groups (n = 176). In the follow-up period, 23% of adults had no infection whereas 6% of the children had no infection (p = 0.002). There was no difference in the frequency of the various haplotypes between adults and children found to be *P. falciparum* + during follow-up (Table 1). Haplotype(s) detected during follow-up were not affected by baseline haplotype(s). For example, of 57 individuals infected solely with EKNG at baseline, 37% were re-infected during follow up with EKNG, 30% with QKNG, 18% with EKNG/QKNG, 7% with ETSR, 2% with EKNG/QTSR (2%), and 7% were not re-infected. This pattern is similar to that seen in overall haplotype infections at baseline and during follow up. Similarly, of 75 individuals with no infection at baseline, 25% of infections detected during follow-up were EKNG, 25% QKNG, 15% EKNG/QKNG and 9% ETSR , and 25% had no infection

**Table 1:** Follow-up period haplotype prevalence in children and adults with infections

	**EKNG**	**QKNG**	**ETSR**	**QTSR**
**Children**	**73%**	**56%**	**18%**	**3%**
**Adults**	**60%**	**60%**	**16%**	**5%**

### Haplotype change over time (n = 176)

Generalized linear models were used to quantify haplotype stability according to baseline haplotype and parasite density, follow-up infection haplotype and parasite density, and age group. Table 2 describes the number of changes in haplotypes according to the number of infections experienced during follow up from weeks 3 through 11 (e.g. change = 1 for an individual with a single EKNG haplotype infection at week 3 and a single QKNG haplotype at week 7). There was no predictive value found of parasite density or individual haplotype at baseline or during follow up for changes in haplotypes over time. Children had significantly more haplotype changes (94%) compared to adults (67%; p < 0.001). An average of 2.8 haplotype changes was observed among children *vs* 1.3 among adults.

**Table 2:** Stability of haplotypes detected during the follow-up period (weeks 3–11)

	**No Change**	**1 Changes**	**2 Changes**	**3 Changes**	**4 Changes**	**5 Changes**	**6 Changes**
**2 Infections**	20 (44%)	25 (56%)					
**3 Infections**	4 (15%)	4 (15%)	0				
**4 Infections**	3 (14%)	16 (59%)	3 (11%)	0			
**5 Infections**	1 (8%)	1(8%)	4 (33%)	4 (33%)	0		
**6 Infections**	0	2 (11%)	4 (22%)	5 (28%)	6 (33%)	1 (6%)	
**7 Infections**	1 (3%)	4 (11%)	4 (11%)	8 (23%)	8 (23%)	8 (23%)	2 (6%)

Number of haplotype changes is displayed by number of infections detected. Individuals who had one or no infections detected during the follow-up period were excluded (n = 40).

### Baseline haplotype and time-to-infection (n = 176)

Kaplan-Meier curves and log rank tests comparing individuals infected at baseline with any haplotype and time-to-infection (with any haplotype) found no differences. Additional analysis grouping those infected with the most prevalent haplotypes (EKNG and QKNG) *vs* less prevalent haplotypes (ETSR and QTSR) also did not demonstrate any difference in time-to-infection (p = 0.37). Thus baseline haplotype infection had no observable effect on subsequent infecting haplotypes or time-to-infection.

### Baseline and follow-up haplotype and parasite density (n = 201)

**Figure 4:**
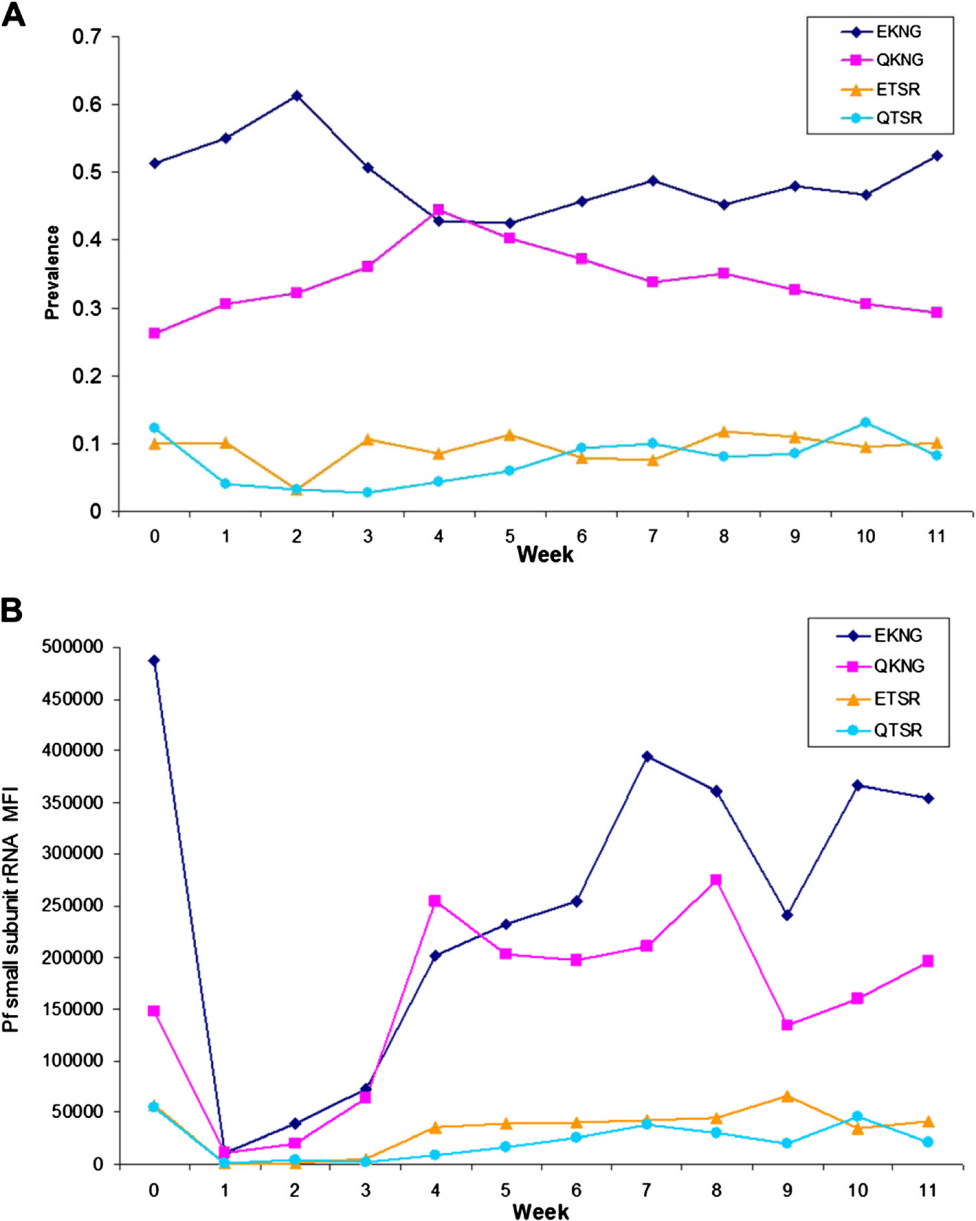
**Haplotype prevalence and weighted density.** Panel **A** illustrates prevalence of the four haplotypes in each week. Panel **B** illustrates the cumulative infecting *P. falciparum* density associated with each haplotype by week for the study population.

Haplotype prevalence by week is displayed in Figure 4A. Each haplotype’s prevalence is relatively stable throughout the study period. EKNG and QKNG exhibit some variability in weeks 4 and 5 as new infections were detected in the population. To visualize the relationship between haplotype and parasite density, each infecting haplotype was adjusted for parasite density (*P. falciparum* small subunit RNA MFI of the LDR-FMA) and totalled by week. If multiple haplotypes were present in a single infection, a proportional parasite density was attributed to each haplotype. For example, if an infection contained similar quantities of QKNG and EKNG determined by allele specific MFI, then 50% of the *P. falciparum* density (measured by *P. falciparum* small unit RNA MFI) was assigned to QKNG and 50% to EKNG. If an infection contained both QKNG and EKNG but the former was predominant, then 75% of the *P. falciparum* density was assigned to QKNG and 25% to EKNG [23]. Figure 4B displays these data and illustrates the gradual resurgence of predominant EKNG and QKNG haplotypes over time, while ETSR and QTSR haplotypes remained low and comparably stable. 

As previously stated, children had higher baseline parasite densities compared to adults. Individuals infected with multiple haplotypes at baseline tended to have higher parasite densities compared to individuals infected with single haplotypes at baseline (MFI 8,140 *vs* 3,578, t = −4.76, p < 0.001; n = 176). Because children had higher parasite densities, they tended to have multiple haplotypes detected (see section regarding symptomatic malaria). However, no differences were observed in baseline parasitaemia according to individual baseline haplotype. During the follow-up period, parasite density remained greater in children than adults (4,106 *vs* 1,824, t = −2.65, p < 0.001). However, baseline haplotypes did not predict re-infection parasite density (in individuals who had both baseline and follow-up period infections). At the first time of re-infection, parasite density was consistently lower than at baseline. The amount of decrease, however, was not related to the baseline density (R^2^ = 0.03).

Individuals with multiple haplotype infections during the follow-up period had greater parasite density compared to individuals with single haplotype infections. QKNG/ETSR, EKNG/ETSR or EKNG/QTSR infections had significantly greater density (MFI range 4,704-8,879) compared to EKNG/QKNG, QKNG, EKNG, ETSR, and QTSR (MFI 572–3,216) (p < 0.001, ANOVA). Using these estimates of parasite density in a generalized linear model controlling for 1) baseline haplotype and density; 2) follow-up infection haplotype and density; and, 3) age, it was found that age and haplotype complexity remained predictors of parasite density (p = 0.081 and 0.024, respectively). Specifically, follow-up infections containing QKNG/ETSR resulted in greatest parasite densities (mean MFI 9,521), but this was not significantly different from the other combination haplotypes (EKNG/ETSR or EKNG/QTSR) exhibiting greater densities than single haplotype infections. With these data, parasite density over time was then examined. Longitudinal models were created to estimate parasite density after first infection during follow-up. It was found that the parasite density was unstable and did not follow an observable trend; there was poor model fit.

### Relationship between baseline variant-specific antibody responses, haplotype-specific infections, and time-to-infection (n = 176)

IgG antibodies directed against MSP-1_19_ (EKNG, QKNG, ETSR, and QTSR) and MSP-1_42_ (EKNG, QKNG, and ETSR) were measured by ELISA and Luminex® multiplex assay using plasma samples obtained at baseline. Additionally, functional MSP-1_19_ IIA (ETSR) was measured. No plasma samples were available from the follow-up period. There was no correlation between MSP-1_19_ IIA (ETSR only) and antibodies to MSP-1_42_ (ETSR; kappa = 0.0131) or MSP-1_19_ (ETSR; R^2^ = 0.0147) measured by serology, as previously demonstrated [18,32].

**Figure 5:**
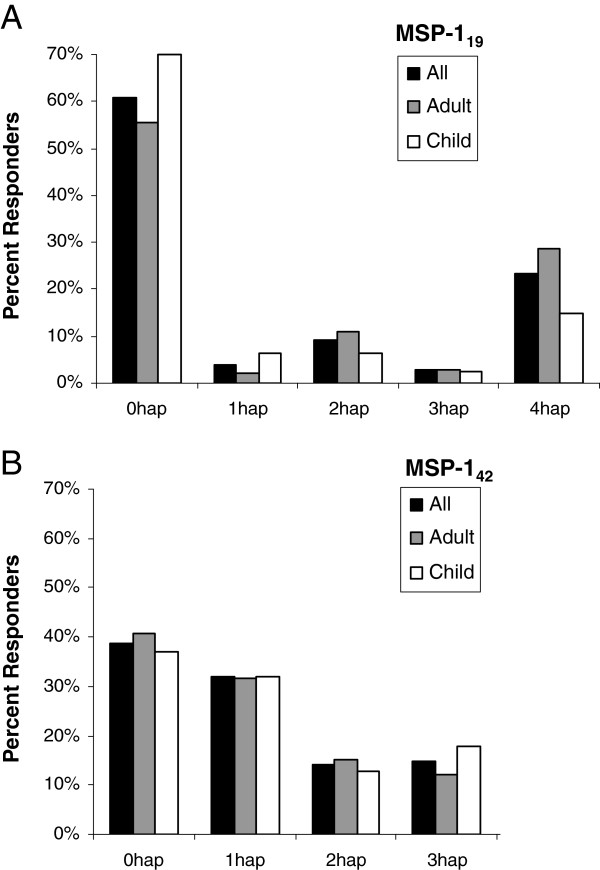
**Distribution of variant-specific antibody responders for MSP-1**_**19**_** and MSP-1**_**42**_** at baseline.** All responders (black columns), adults (grey columns) and children (white columns) frequencies are displayed for either MSP-1_19_ variants (panel **A**) or MSP-1_42_ variants (panel **B**). Note that MSP-1_42_ QTSR was not tested, thus the maximum number of antigens participants could respond to is three. No statistical difference between frequency of adult and children was detected for any group.

**Figure 6:**
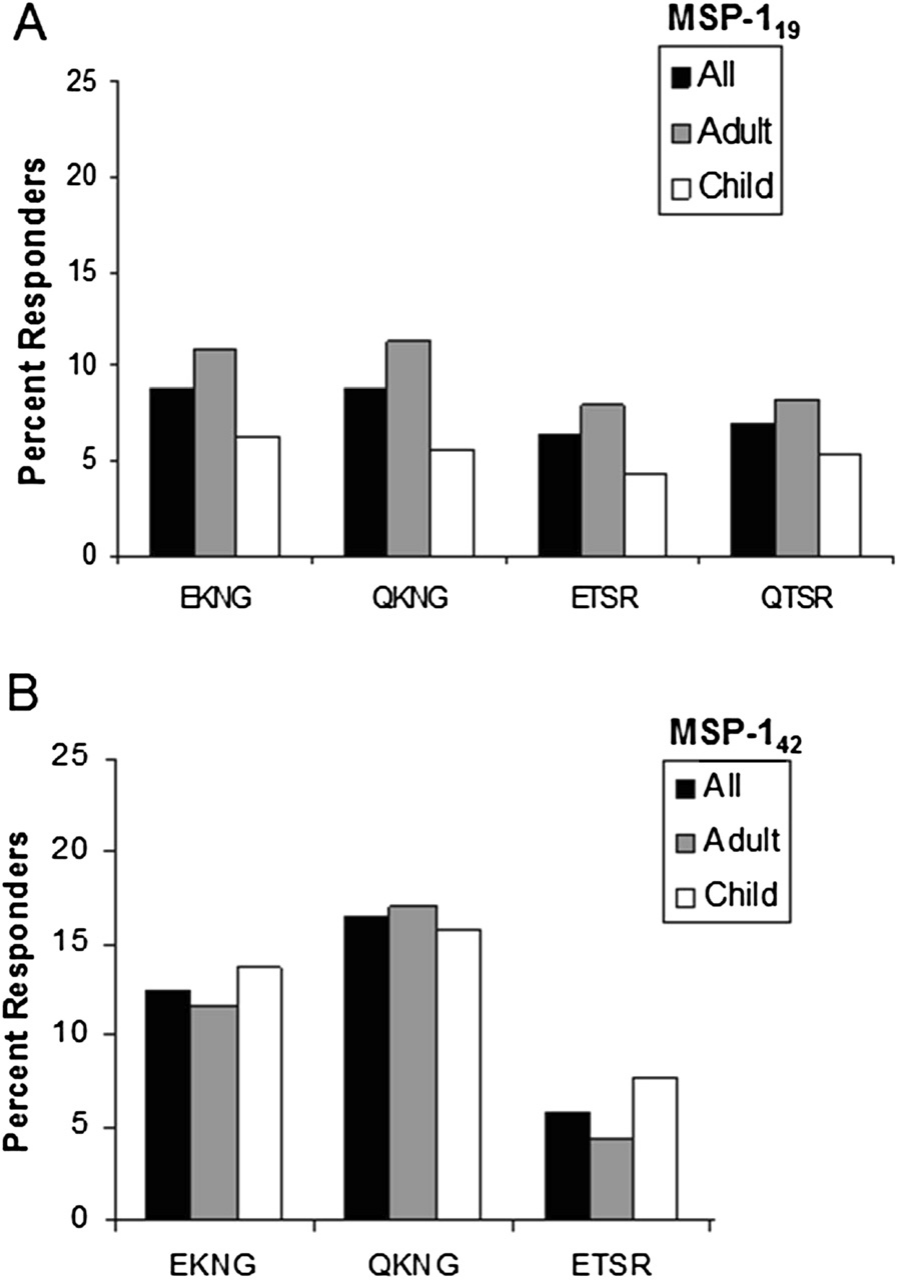
**Distribution of variant-specific antibody responders for MSP-1**_**19**_** and MSP-1**_**42**_** at baseline stratified by variant.** All responders (black columns), adults (grey columns) and children (white columns) frequencies are displayed for either MSP-1_19_ variants (panel **A**) or MSP-1_42_ variants (panel **B**). MSP-1_42_ QTSR was not examined. No statistical difference between prevalence in adult and children was detected for any variant.

Figure 5A and 5B illustrate the proportion of serologically measured variant-specific antibody responders to recombinant MSP-1_19_ and MSP-1_42_. First, the frequency of an individual variant response was examined. Interestingly, many participants had no detectable variant-specific antibodies (approximately 40% and 60% of participants did not respond to MSP-1_19_ or MSP-1_42_). There were no statistical differences in percent responders detected between adults and children or between responders with antibodies to multiple variants *vs* no variants. With regard to the frequency of variant-specific responses among those individuals who were responders, there was no statistical difference detected among variants of MSP-1_19_ and MSP-1_42_ or between adults and children (Figure 6A and 6B). The results were consistent whether antibodies against MSP-1_19_ variants were measured by ELISA or antibodies against MSP-1_42_ variants were measured by Luminex® multiplex assay. 

**Figure 7:**
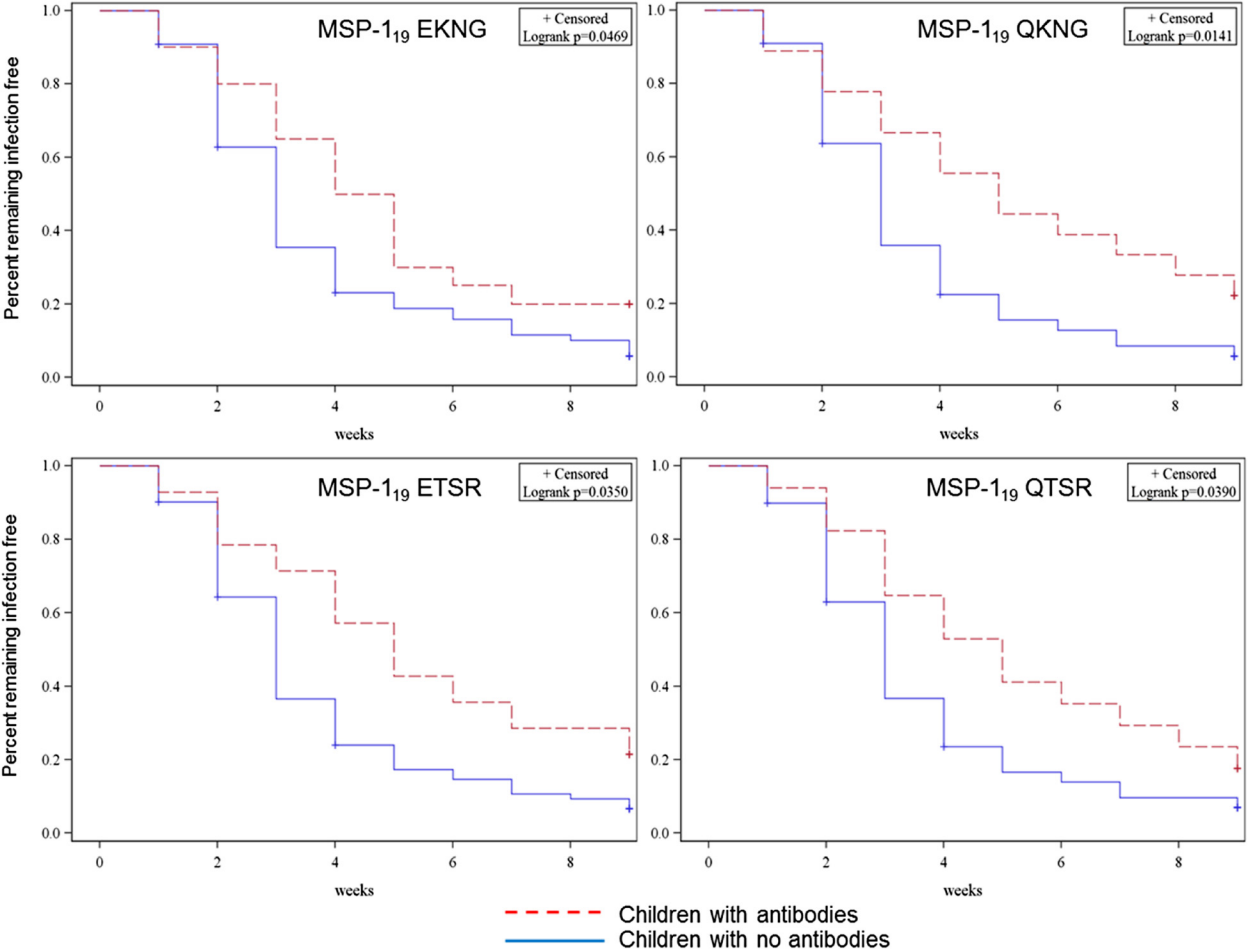
**Kaplan-Meier curves illustrating delay in time-to-infection for children with MSP-1**_**19**_** variant-specific antibodies at baseline.** The four Kaplan-Meier curves show the percent of children remaining malaria infection free (measured by BS) on the Y axis over time (weeks of follow up, X axis) stratified by whether the children had MSP-1_19_ variant-specific antibodies (EKNG, QKNG, ETSR or QTSR) at baseline (week 0). Children with antibodies (responders) are represented with a dashed red line, and those with no antibodies are represented with a solid blue line. Children with antibodies against the MSP-1_19_ variants had a delayed time-to-infection compared to those with no antibodies.

Children had a shorter time-to-infection compared to adults, presumably due to less well developed clinical immunity (average week of infection for children was 5.3 (BS) and 2.5 (PCR/LDR-FMA) *vs* adults 7.4 (BS) and 5.0 (PCR/LDR-FMA)). Children who were MSP-1_19_ variants responders at baseline had a delayed time-to-infection as measured by BS compared to children with non-responders (Figure 7). Children with high-level antibodies (upper tercile) to MSP-1_42_ FUP/EKNG (wilcoxan test p = 0.035; log-rank p = 0.057) or upper two tercile antibodies to MSP-1_42_ FVO/QKNG (log-rank p = 0.0324), had delayed time-to-infection as measured by BS, whereas those with upper tercile antibodies to MSP-1_42_ 3D7/ETSR demonstrated no statistically significant delay to infection (log-rank p = 0.3760). Adults with antibodies to MSP-1_19_ or MSP-1_42_ variants had no delay in time-to-infection compared to non-responders. When time-to-infection was measured by PCR/LDR-FMA, similar trends were observed but statistically significant delays in time-to-infection were only observed in children responders to MSP-1_19_ ETSR (log-rank p = 0.0223) and QTSR (log-rank p = 0.0350) but not QKNG (log-rank p = 0.0617) or EKNG log-rank (p = 0.3692). Children with antibodies to MSP-1_42_ FUP/EKNG (log-rank p = 0.0143) but not 3D7/ETSR (log-rank p = 0.8) or FVO/QKNG (log-rank p = 0.1) had delayed time-to-infection compared to children with no variant-specific antibodies. Adults with antibodies to MSP-1_19_ or MSP-1_42_ variants had no delay in time-to-infection compared to non-responders. Children with antibodies to multiple variants had no difference in time-to-infection compared with children with antibodies to one variant. No change in time-to-infection (measured by BS or PCR/LDR-FMA) as related to MSP1_19_ IIA (ETSR) responses was observed in children or adults.

One of the goals was to determine if variant-specific antibodies to the C-terminal 19 kDa region of MSP-1 present at baseline were predictive of protection from subsequent haplotype-specific infection. 176 participants were characterized as responders or non-responders for serologic responses to each antigen tested at baseline. Chi-squared tests for univariate analysis did not demonstrate any significant relationships between baseline variant-specific serology or functional antibodies and baseline or follow-up infection haplotypes. Logistic regressions controlling for age, baseline haplotype and parasite density also did not reveal any significant associations between baseline variant-specific serology or functional antibodies and infecting haplotypes (baseline or follow-up period infections). Variant-specific antibodies did not have a protective or detrimental effect on subsequent haplotype-specific infection (or lack of infection). In summary, children with variant-specific MSP-1_19_ antibodies demonstrated delayed time-to-infection, but follow-up infection haplotype bore no relationship to baseline MSP-1_19_ variant-specific antibodies.

### Characteristics of participants who developed symptomatic uncomplicated malaria infections during the follow-up period

**Figure 8:**
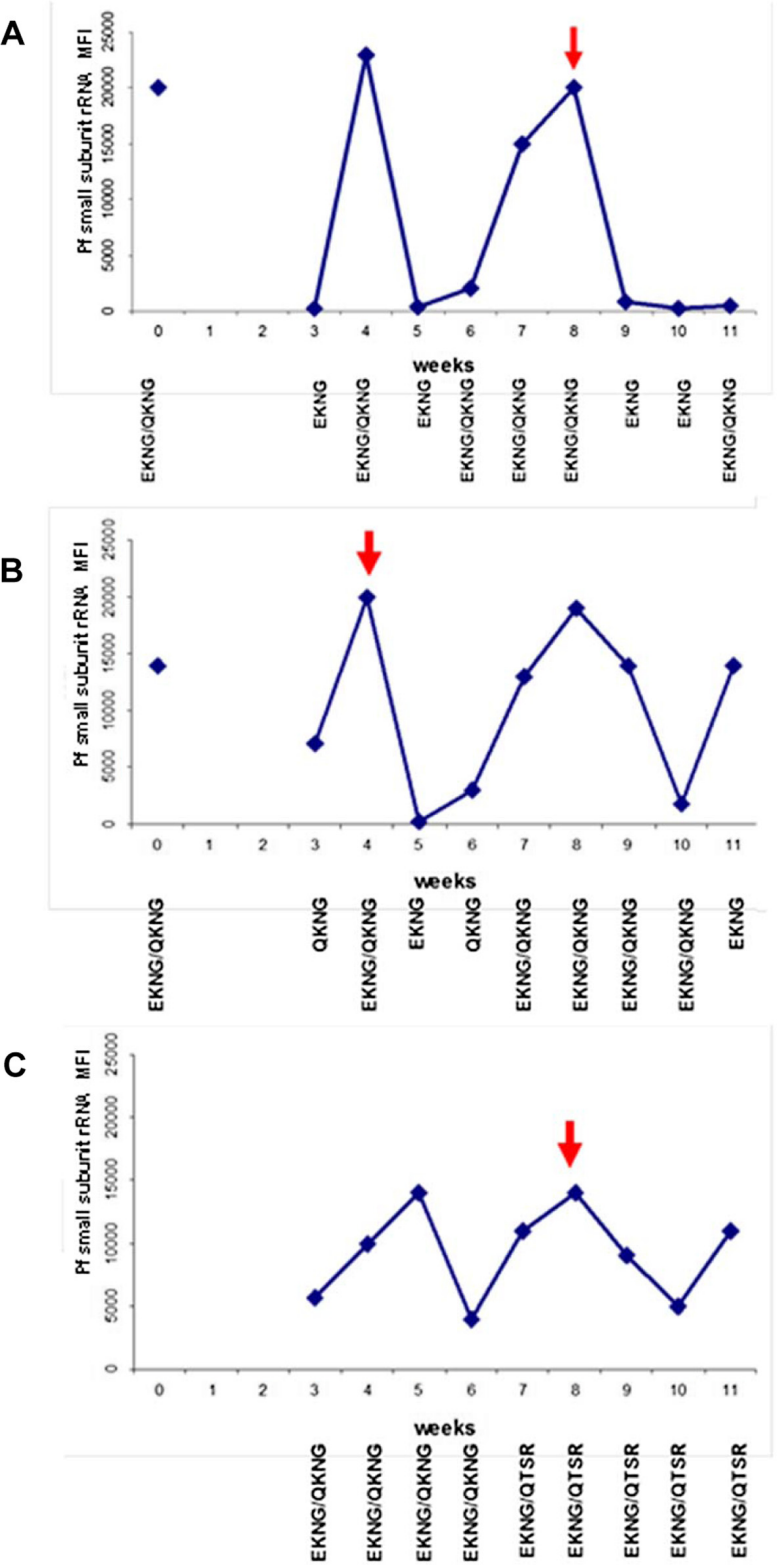
**Examples of detected infections for three symptomatic children over the study period demonstrating *P. falciparum *density and haplotypes detected.** The red arrow denotes the week each child developed symptomatic clinical malaria and was retreated with Coartem®. The X axis denotes the week of the study and the Y axis denotes *P. falciparum* density by MFI. In Panel **A**, *P. falciparum* infection was detected several weeks prior to symptoms. With low parasitaemia, only a single haplotype (EKNG) was detected. It may be that amplifying the Q allele was below the threshold of detection with low parasitaemia. In Panel **B**, Coartem® administration was followed by a rapid decline of detectable parasitaemia, but increases in parasitaemia were seen in weeks 7–9. Panel **C** shows infections in weeks 3–6 with ENKG/QKNG haplotypes. In week 7, a new haplotype QTSR was detected prior to the development of clinical malaria.

During the 11 weeks of follow up after baseline Coartem administration, 18 individuals developed febrile malaria infections (axillary temperature >37.8°C and parasitaemia) requiring retreatment with CoArtem®. Seventeen individuals were children (mean age 7.3 years, range 2.1-11.5 years). Six of these 18 individuals had no infection at baseline. The predominant haplotypes at baseline of the other 12 individuals were EKNG (four), EKNG/QKNG (five), and EKNG/ETSR (three), which is a haplotype prevalence similar to that observed in the general population. At the time of symptomatic infection, five individuals were infected with EKNG/QKNG haplotypes, five with EKNG/QTSR, two with EKNG, and one individual for each of the remaining haplotypes (EKNG, QKNG/ETSR, QKNG/QTSR, EKNG/ETSR, ETSR, QTSR). This haplotype frequency again reflects the general population haplotype distribution during the follow-up period. All symptomatic individuals had asymptomatic infections prior to malarial disease (minimum one week prior, maximum seven weeks). Fifteen had detectable infections one to two weeks after CoArtem® retreatment (measured by PCR/LDR-FMA), but the parasite densities were lower compared to parasite densities associated with symptoms. Five individuals had a decrease in detectable parasite density for one to two weeks after medication, but then an increase in parasite density in the subsequent weeks (an example is shown in Figure 8A). Nine symptomatic individuals had new haplotypes detected one to two weeks prior to symptoms (an example is shown in Figure 8B). Nine study participants had all haplotypes present at some point during the follow-up period (an example is shown in Figure 8C). With respect to the presence or magnitude of antibody responses of these symptomatic individuals, no statistical difference was detected between this group and those who did not have disease or age-matched asymptomatic individuals. Of note, none of the symptomatic individuals had any MSP-1_19_ IIA detectable at baseline compared to 6% of the general population. Over the entire follow-up period, 17 symptomatic individuals had more than one haplotype present in their infections, with an average of 3.04 different haplotypes during the follow-up period compared to 2.72 among age-matched, asymptomatic individuals (p = 0.185). Symptomatic individuals tended to have higher *P. falciparum* densities at baseline and during the follow-up period compared to age-matched, asymptomatic individuals, but this was not statistically significant (p = 0.08 and p = 0.67, respectively).

## Discussion

Antigenic polymorphism is considered a significant confounder in the development of antibody-mediated protection against blood stage *P. falciparum* in the context of naturally acquired immunity and malaria vaccine development. The goal of this study was to determine whether variant-specific antibodies to MSP-1_19_ were associated with haplotype-specific protection in a cohort of Kenyan adults and children who participated in a treatment time-to-infection study. These data showed that i) baseline infecting MSP-1_19_ haplotype had no effect on the subsequent infecting haplotypes; ii) variant-specific IgG antibodies measured serologically and functionally had no association with follow-up infecting haplotypes or density; iii) variant-specific antibodies correlated with delayed time-to-infection among children but not adults; and, iv) variant-specific antibodies were associated with protection in a haplotype-transcending manner. Considered together, these data found no evidence for haplotype-specific immunity to MSP-1_19_ in this study of naturally infected individuals living in a malaria holoendemic region.

EKNG and QKNG were the most prevalent MSP-1_19_ haplotypes in this population and region of western Kenya in 2003 when this study was conducted. From other surveys, it has been found that this distribution of MSP-1_19_ haplotype distribution pattern is stable (Yeo, unpublished). Takala *et al.* found comparable results with EKNG and QKNG being the most prevalent haplotypes in Mali from 1999 to 2001 [33]. Others have shown similar dominances of EKNG and QKNG in Kenya, Brazil, Vietnam, Thailand, Tanzania and Vanuatu [24,34-36].

It was found that within a single individual, infecting haplotypes frequently changed from week to week. This could be due in part to sampling effect. A finger-prick blood sample does not accurately reflect total body haplotype prevalence or parasitaemia. Additionally, this study showed that with low parasitaemia haplotype detection may not be optimal (Figure 8). In several studies, parasite densities and parasite genotypes varied significantly within a 24-hour time period and over days to weeks [37-40]. Furthermore, venous blood may reflect different densities than finger-prick blood as the latter would be expected to have a greater frequency of capillary-sequestered infected erythrocytes. Examining weekly infections may heighten the variability detected, but with repeated measures in 176 participants over a 12-week study period, overall trends should persist. To this end, it was found that haplotype complexity was associated with *P. falciparum* density and possibly symptomatic infections. This contrasts with others’ finding that complexity of infection was associated with increased age and decreased frequency of symptomatic infection [33]. An important point in this regard is that children had higher parasite densities than adults, and this most likely led to better detection of multiple haplotypes.

Analysis of serologic responses was performed using recombinant MSP-1_19_ (four variants) and MSP-1_42_ (three variants). Variability in protein folding and expression systems used to produce these products, e g, yeast and *E. coli*, and serology techniques (traditional ELISA *vs* Luminex® multiplex) could account for differences in determining antibody responders *vs* non-responders. Nevertheless, these data indicated that both approaches produced a similar overall result — no discernible variant-specific immune correlation, consistent with the notion that variant-specific antibodies cross react with heterologous variants. Using immunodepletion assays, Zakeri *et al.* found evidence of antibody cross-reactivity among several MSP-1_19_ variants, consistent with these findings [36].

A significant limitation to the approach of detecting the infecting haplotype is the assumption made to assign two haplotypes to an infection that contained all four alleles. Although in most cases a predominant haplotype could be differentiated from a minor haplotype with the MFI of each detected allele, it is not certain that only two haplotypes were present [23]. For example, if an infection composed of all four alleles had higher MFIs for Q > E and KNG > TSR, the haplotypes would be assigned as QKNG and ETSR. However, it is possible that the individual was actually infected with QKNG, EKNG and ETSR. The only way to definitively determine this would be to have a larger blood volume and clone and sequence multiple PCR products, an approach which was not feasible for this study. Other methods such as pyrosequencing are advantageous in that direct sequencing of amplicons is possible.

This study had limited power to detect associations between antibody responses and infecting haplotypes in the context of susceptibility to symptomatic malaria. The 18 individuals who developed clinical malaria during the follow-up period did not have a discernible variant-specific antibody pattern. They did, however, lack MSP-1_19_ IIA antibodies, which has previously been shown to increase with haplotype-specific (ETSR) infection [29]. Interestingly, 15 of these symptomatic individuals had *P. falciparum* detectable by PCR at least one week after treatment. Although this study was not designed to examine the efficacy of Coartem® treatment, previous observations demonstrate that parasites are cleared from the blood within 48 hours [41]. Although the possibility that detection of *P. falciparum* after treatment resulted from residual *P. falciparum* DNA cannot be excluded, it is most likely that detection resulted from the progression of pre-existing liver stage *P. falciparum* to the blood stage as Coartem® does not eliminate the former. Inadequate adherence to treatment regimen and/or lack of food intake with medication consumption could also result in incomplete parasite clearance [42,43], but is unlikely with this study as all six doses of Coartem were directly observed by project staff. All but one participant with symptomatic malaria during the follow-up period had infections containing three or more MSP-1_19_ alleles. Increased COI may be associated with increased risk of symptomatic malaria, as has been observed previously [44,45]. Malaria transmission intensity and seasonality may also affect COI, but this study was not designed or powered to detect this association.

## Conclusion

Healthy asymptomatic children and adults living in a holoendemic malaria region displayed no MSP-1_19_ variant-specific antibody protection (measured serologically or functionally) against haplotype-specific infections regardless of age or parasite density (baseline or follow-up infection). The infecting haplotype frequency reflected the population haplotype prevalence even after drug clearance. There was no discernible relationship between variant-specific antibody responses and haplotype-specific infections. Variant-specific antibody responses and occurrence of malaria disease was not evaluated in this study but needs to be addressed in order to better inform vaccine development.

## Abbreviations

MSP: Merozoite Surface Protein; BS: Blood smear; LDR-FMA: Ligase Detection Reaction-Fluorescent Microsphere Assay; MSP-1_19_ IIA: MSP-1_19_ Invasion Inhibitory Antibodies; COI: Complexity of infection.

## Competing interests

The authors declare that they have no competing interests.

## Authors’ contributions

AED, AMM, JWK conceived and designed the experiments. AED, CTY, RJK performed the experiments. AED, DJT analysed the data. CAL, DLN, BSC contributed reagents. AMM, POS, JV managed field study participant involvement and sample collection. All authors prepared the manuscript. All authors read and approved the final manuscript.
